# Understudied Factors Influencing Fc-Mediated Immune Responses against Viral Infections

**DOI:** 10.3390/vaccines7030103

**Published:** 2019-08-30

**Authors:** Sai Priya Anand, Andrés Finzi

**Affiliations:** 1 Centre de Recherche du CHUM, Montreal, QC H2X 0A9, Canada; 2 Department of Microbiology and Immunology, McGill University, Montreal, QC H3A 2B4, Canada; 3 Department of Microbiologie, infectiologie et immunologie, Université de Montréal, Montreal, QC H2X 0A9, Canada

**Keywords:** antibodies, Fc-mediated effector functions, ADCC, ADCP, ADCML, viruses

## Abstract

Antibodies play a crucial role in host defense against viruses, both by preventing infection and by controlling viral replication. Besides their capacity to neutralize viruses, antibodies also exert their antiviral effects by crystallizable fragment (Fc)-mediated effector mechanisms. This involves a bridge between innate and adaptive immune systems, wherein antibodies form immune complexes that drive numerous innate immune effector functions, including antibody-dependent cellular cytotoxicity, antibody-dependent complement-mediated lysis, and antibody-dependent phagocytosis. Here, we review certain mechanisms that modulate these antibody-mediated effector functions against virally infected cells, such as viral glycoprotein shedding, viral glycoprotein internalization, antibody cooperativity, and antibody glycosylation. These mechanisms can either protect viral replication or enhance infected cell clearance. Here we discuss the importance of these understudied factors in modulating Fc-mediated effector functions.

## 1. Background

Antibodies carry out a multitude of preventative and therapeutic antiviral activities. They can either neutralize viral particles via their antigen binding fragment (Fab) domains or interact with innate immune receptors, such as crystallizable fragment-gamma receptors (FcγRs), via their crystallizable fragment (Fc) domains. The diverse FcγR-mediated mechanisms to eliminate targets exposing vulnerable antigens include antibody-dependent cellular cytotoxicity (ADCC), antibody-dependent complement-mediated lysis (ADCML) and antibody-dependent cellular phagocytosis (ADCP). ADCC is mediated by effector cells, such as natural killer (NK) cells, neutrophils and monocytes, that release cytotoxic molecules to eliminate virally infected cells [1]. In humans, this response is initiated by the Fc regions of IgG1 or IgG3 subtypes that bind the activating FcγRIIIa (CD16a) receptor on effector cells [2]. This is followed by the cross-linking of the FcγRs, resulting in intracellular signaling via immunoreceptor tyrosine-based activation motifs to release granzyme B and perforin granules [3]. Granzyme B causes DNA fragmentation and apoptosis of the target cell [4]. Additionally, signaling via CD16a also leads to the secretion of several cytokines and chemokines, including IFN-γ, TNF, and β-chemokines that inhibit viral spread [5,6,7]. ADCP is carried out by monocytes, macrophages, neutrophils, and eosinophils. These phagocytes can be engaged by either complement receptors and/or FcγRs, including FcγRIIa (CD32a) and FcγRI (CD64) [8,9]. In addition to clearing antibody-opsonized targets, phagocytosis and signaling via FcγRs also lead to the secretion of antiviral cytokines [10]. Finally, ADCML is initiated by the interaction of the IgG Fc with the complement protein C1q to drive direct cytotoxicity, as well as other immunoregulatory functions involving enhancement of phagocytosis and stimulation of antigen-presenting cells [11,12]. These antibody-mediated effector functions have been implicated in the protection and control against many viruses, including influenza viruses [13], Ebola virus (EboV) [14,15], and the human immunodeficiency virus (HIV-1) [16].

In the context of HIV-1 infection, the use of broadly neutralizing antibodies (bNAbs) has been well studied and is currently being evaluated to both prevent and control infection (NCT03571204) [17,18,19,20,21]. These are HIV envelope (Env)-specific antibodies that exhibit high potency and neutralization breadth across different clades of HIV-1. Numerous studies have passively administrated bNAbs in non-human primate models to protect from chimeric simian-human immunodeficiency (SHIV) challenges and control viremia [17,22,23]. Interestingly, recent studies have also suggested that beside the neutralization activity of bNAbs, Fc-mediated effector responses, such as ADCC, are important in preventing and controlling viral replication in vivo [24]. Diminishing the FcγR binding of bNAbs by introducing leucine-to-alanine substitutions (L234A/L235A or LALA) in their Fc regions significantly reduced the protection from either high-dose or multiple low dose SHIV challenges in rhesus macaques [25,26]. Similarly, studies using bNAbs with glycine to arginine and leucine to arginine substitutions in their Fc regions (G236R/L328R or GRLR) to diminish Fc-dependent functions reduced both the bNAb capacity to block viral entry and clear HIV-1-infected cells in a humanized mice model [24,27,28]. Interestingly, a recent study found that for some bNAbs, such as PGT121, neutralization alone might be sufficient to protect from SHIV-infected cell challenges in rhesus macaques. This conclusion was made by comparing PGT121 and its LALA counterpart, and also by depleting NK cells, both of which had no effect on protection [29]. Despite this study, there are still numerous lines of evidence suggesting the importance of Fc-mediated effector functions in eliminating HIV-1-infected cells [30]. Other studies have shown that ADCC activity of HIV-1-specific antibodies has been positively correlated with slower disease progression [31,32]. Infected individuals harboring higher titers of ADCC-inducing antibodies were shown to have lower viral titers and higher CD4+ T cell counts [33,34]. Lastly, antibodies with ADCC activity, in the presence of low plasma IgA Env-specific antibodies, correlated with decreased HIV-1 acquisition in the partially successful HIV-1 RV144 vaccine trial [35].

Numerous factors govern the magnitude of antibody-mediated effector functions against virally infected cells, and the majority of these are dependent on the ability of immune complex formation and the efficiency with which effector cell are activated. It has been demonstrated that the degree of antibody binding to antigens correlates with ADCC susceptibility, with lack or excess binding disrupting the ability of immune complexes to engage effector functions [36,37]. However, antigen binding per se does not always trigger ADCC responses [38,39], proper engagement and clustering of FcγRs is required to activate effector cells [40]. Since the affinity of antibodies for the majority of FcγRs, including FcγRIIa and FcγRIIIa, is low and in the micromolar range, only multivalent antibody-antigen immune complexes can exert enough avidity to cluster and activate them [1,2,8,41]. Similarly, the stoichiometry and the orientation of the antibodies bound to the antigen, which dictate the accessibility of Fc domains to engage FcγRs, appear to play an important role [42]. FcγR polymorphisms in humans that have greater Fc binding affinity can also influence the occurrence of infection or disease progression of viral infections, including HIV-1 [43,44]. Overall, the level, specificity, isotype, and subclass of antibodies are characteristics that dictate the balance between Fc-mediated protection and disease progression.

Despite the recent surge in the study of Fc-mediated effector functions in controlling viral infections, there remain some understudied parameters. These include viral glycoprotein shedding that can redirect humoral immune responses from infected to uninfected cells, glycoprotein internalization that can modulate immune complex formation and the ability of different families of antibodies to synergize for effector cell activation, as well as antibody glycosylation. This review summarizes these mechanisms with a focus on HIV-1, while drawing parallels with other viruses. Finally, the consequences of these mechanisms in determining the efficacy of Fc-mediated effector responses and their implication for vaccine design and therapeutics are discussed.

## 2. Viral Glycoprotein Shedding

The location and presence of immune complexes dictate which target cells are subjected to effector responses. Redirection of humoral immune responses is possible when viral glycoproteins are either shed or secreted and act as antigen decoys or ‘antibody sinks’ [45]. Glycoprotein shedding or secretion from infected cells can occur either by spontaneous dissociation, transcriptional and translational editing, or the activity of sheddases, such as metalloproteinases [45]. This strategy of antigenic subversion by soluble glycoproteins to divert immunomodulatory effects from productively infected cells has been described for many enveloped viruses, including HIV-1, respiratory syncytial virus (RSV) and EboV, which are discussed below.

The HIV-1 envelope glycoprotein (Env) trimer is derived from the proteolytic cleavage of a trimeric gp160 precursor [46,47] and is composed of gp120 exterior and gp41 transmembrane subunits. Since there are non-covalent interactions between the two subunits, this results in the spontaneous dissociation of gp120 from gp41, known as gp120 shedding, from the surface of productively infected cells [48,49,50]. Consequently, significant levels of soluble gp120 can be found circulating in the blood and tissues of HIV-infected individuals [51,52,53]. Shed gp120 can interact with CD4 receptors on the surface of uninfected bystander cells [54]. It is now well established that the interaction of gp120 with CD4 is critical for the exposure of highly conserved epitopes for ADCC-mediating antibodies [55,56]. Importantly, CD4-induced ADCC-mediating antibodies are naturally present in the sera of HIV-infected individuals, and HIV-1 has evolved to protect infected cells from being recognized by these antibodies [57,58]. Thus, binding of shed gp120 to CD4 receptors on uninfected CD4+ T cells redirects ADCC responses away from the productively infected cells to bystander cells. This phenomenon, reported by Richard et al., results in the elimination of uninfected bystander cells [54]. The preferential binding of non-neutralizing, ADCC-mediating antibodies to bystander cells was confirmed by Bruel et al. [59], and in vivo using a humanized mice model by Horwitz et al. [27]. The in vivo effects of shed gp120 binding to the surface of bystander lymphocytes has been suggested to serve as effective targets that contribute to decreased CD4+ T cell counts in HIV-1-infected individuals [54,60]. Moreover, in vitro ADCC assays can be greatly influenced by the redirection of ADCC towards uninfected CD4+ T cells [61].

RSV has two major glycoproteins, the attachment (G) and fusion (F) glycoproteins. In addition to the full-length membrane bound G, a soluble form (sG) is expressed and released from infected cells. sG is produced from an alternative translation initiation that sensitizes the RSV G to peptidase cleavage [62,63]. Studies by Bukreyev et al. examined the role of sG in vivo using a mouse model and observed that sG protected RSV replication by occupying neutralizing antibodies. Additionally, by diverting G- or F-specific antibody responses, sG also inhibited Fc-mediated immune responses by macrophages and complement [64,65]. Similarly, the EboV glycoprotein (GP) gene also undergoes transcriptional editing to encode for a secreted dimeric GP (sGP) protein [66,67]. Additionally, some studies have observed the involvement of tumor necrosis factor α-converting enzymes (TACE) in the proteolytic cleavage and shedding of EboV GP from the infected cell surface [68]. The EboV sGP has also been implicated in impeding immune-mediated clearance of EboV infection [69,70]. Despite structural differences between GP and sGP, with GP being trimeric and sGP forming homodimers, they share some cross-reactive epitopes, including those of some non-neutralizing antibodies, that sGP can compete for [71,72]. Thus, sGP could divert Fc-mediated effector functions away from infected cells; this interesting hypothesis remains to be formally tested. Further supporting this idea, studies using mice models have shown that B-cell responses can be biased towards epitopes that are shared between sGP and GP, rendering antibody responses susceptible to sGP competition [73,74]. Altogether, these findings highlight the relevance of measuring the impact of soluble form of Envs on Fc-mediated effector functions both in vitro and in vivo.

## 3. Viral Glycoprotein Internalization

The formation of stable immune complexes that can be presented to effector cells is another significant factor dictating the efficiency of Fc-mediated immune responses against different viruses. This parameter can be modified in many ways, including viral envelope protein internalization, a characteristic observed with most viruses, including HIV-1 [75]. The cytoplasmic domains of many viral envelopes contain endocytic motifs that are mainly tyrosine-based (Y) or dileucine-based (LL) motifs. These are recognized by adaptor complexes (AP), which concentrate proteins within clathrin-coated vesicles [76]. The advantages of glycoprotein endocytosis for viruses vary from promoting replication to hiding from immune responses [77]. For example, endocytosis of Env from some viruses, such as Nipah virus [78] and herpes simplex viruses [79], has been implicated in the regulation of cell-cell fusion for spreading infection.

Lentiviral Env glycoproteins, including HIV-1, have long cytoplasmic domains that are involved in the regulation of Env trafficking and contain several trafficking signals. These include the membrane-proximal tyrosine-based sorting motifs (YXXφ) that interact with AP-2 and dileucine motifs that interact with AP-1 [80,81,82]. The endocytosis and recycling of HIV-1 Env has been suggested to be an essential mechanism in Env incorporation within viral particles [83]. Furthermore, a study by von Bredow et al. introduced mutations in these endocytic motifs and showed increased cell-surface expression of Env, which correlated with increased ADCC responses. Consequently, the authors suggested a role of Env endocytosis in protecting HIV-1 infected cells from ADCC by minimizing Env exposure [84]. Internalizing envelope glycoproteins from the cell surface to avoid humoral immune responses has been observed for other viruses, including equine herpesvirus-1 [85] and pseudorabies virus [86], wherein both downregulate their envelope to protect infected cells from ADCML.

In addition to spontaneous envelope endocytosis, previous reports have demonstrated that antibody binding can induce internalization of the antibody-Env immune complexes. This mechanism of internalization can result from cross-linking of glycoproteins [87] or depend on endocytic motifs discussed above. Accelerated envelope internalization upon antibody binding from the infected cell-surface has been observed with several viruses, including herpesviruses [86,88], RSV [87], feline coronavirus [89], and HIV-1 [90]. In these studies, Fc-effector functions were reduced upon the addition of specific antibodies that could induce glycoprotein internalization, such as a 50% reduction in ADCML of pseudorabies virus-infected monocytes [86]. Similarly, reduction of antibody-induced Env internalization using a dynamin inhibitor enhanced the susceptibility of HIV-1-infected cells to ADCC [90]. Interestingly, our group observed that antibody-bound Env proteins are internalized in an Env conformation specific manner [90]. We found that bNAbs preferentially recognizing the ‘closed’/ State 1 Env conformation triggered rapid Env internalization when compared to non-neutralizing antibodies that preferentially bound the ‘open’/State 2/3 Env conformation. Overall, these results suggest that any parameter modulating Env internalization can potentially impact Fc-mediated effector functions.

## 4. Antibody Cooperativity

The cancer field is leading the way in the combination of monoclonal antibodies (mAbs) that target two or more non-overlapping antigenic epitopes to enhance innate anti-tumor effector immune responses [91,92,93]. Current antiviral strategies also include incorporating mAbs that either target highly conserved epitopes [94] or combinations of two or more mAbs targeting various epitopes [95]. Some examples of antiviral combination therapies using mAb cocktails include SYN023 and CL184 against rabies virus variants [96,97]. Regarding HIV-1, recent therapeutic designs for passive immunization are utilizing combinations of bNAbs to suppress viral rebound. A phase 1b clinical trial carried out by Mendoza et al., used two bNAbs targeting different epitopes, the CD4 binding site (3BNC117) and the base of the V3 loop (10–1074), during antiretroviral treatment interruption and observed suppression of viral rebound without the development of resistant viruses for up to thirty weeks [98]. Interestingly, previous studies have highlighted the importance of 3BNC117 to engage with FcγRs to protect against infection, control viral loads in a humanized mice model and also accelerate the clearance of infected cells [28]. Therefore, combinations of bNAbs not only aid in preventing the emergence of antibody-resistant viral variants, but could possibly also aid in clearance of infected cells by antibody-mediated effector functions. Further studies to tease out the relative contribution of neutralization to prevent infection versus elimination of infected cells by Fc-mediated effector functions of bNAbs are warranted. Additionally, we have recently reported another aspect of antibody cooperativity to clear HIV-1-infected cells. We observed that the Fc regions of two families of antibodies against HIV-1 Env are required to optimally engage FcγRIIIa and mediate robust ADCC [40]. Interestingly, a naturally occurring dimeric form of a potent neutralizing anti-HIV-1 antibody, 2G12, containing four Fabs and two Fc regions, was previously shown to be more potent at mediating ADCC than its monomeric counterpart [99]. Altogether, these studies suggest that multiple antibodies opsonized to the infected cell surface enhance the clustering and cross-linking of FcγRs to activate effector cells and might have beneficial outputs for the host [100].

Similarly, a recent study by He et al. showed that the interactions among Abs targeting different epitopes in polyclonal responses can significantly modulate the extent of Fc receptor activation against the influenza A virus. Herein, the authors demonstrated a coordination between neuraminidase-binding Abs with hemagglutinin stalk-binding Abs to induce ADCC in an additive manner [101]. Similarly, combinations of mAbs targeting non-overlapping epitopes against EboV have reduced viral loads and provided significant protection against EboV disease (EVD) in mouse [102,103], guinea pig [104], and non-human primate studies [105,106]. Subsequently, treatments that were approved for EVD treatment in the 2018 EboV outbreak included the ZMapp [107] and REGN-EB3 [108] mAb cocktails. ZMapp consists of two neutralizing antibodies and a non-neutralizing antibody, 13C6. Strikingly, the removal of 13C6 from this cocktail has shown to decrease protection from EboV, suggesting that the Fc-mediated effector functions of 13C6 are necessary [105,109,110,111]. Finally, studies by Pal et al. used combinations of antibodies against chikungunya virus (CHIKV) to limit viral resistance and to provide in vivo protection against CHIKV infection [112]. Importantly, the therapeutic activity of one of these CHIKV antibodies was shown to depend on FcγR interactions. Thus, these evidences support the importance of polyclonal humoral responses, with antibodies targeting multiple specificities, against viral infections.

## 5. Antibody Glycosylation

As previously discussed, amino acid mutations within the Fc portion of Abs can impact their ability to engage with FcγRs. Some of these modifications include the LALA and GRLR mutations to decrease FcγR binding or the GASDALIE (G236A/S239D/A330L/I332E) mutation to increase FcγR binding [113]. Moreover, other mutations in the Fc domain include the YTE (M252Y/S254T/T256E) and LS (M428L/N434S) substitutions to enhance interactions with the neonatal Fc receptor and consequently, increase the serum half-life of antibodies and extend their therapeutic effect [114,115]. Interestingly, the glycosylation diversity of the N-acetylglucosamine and mannose core at position asparagine 297 in the C_H_2 domain is another Fc domain modification that regulates the efficiency of Fc-FcγR interactions [116]. In humans, there exist up to 144 possible IgG glycan and subtype combinations, due to the presence of a terminal galactose, fucose, N-acetylglycosamine [GlcNAc], or sialic acid. These glycans have been shown to influence the binding to FcγRs on effector cells and, consequently, alter antibody functionality [117]. The presence of fucose decreases the affinity of the Fc domain for FcγRIIIa and thereby reduces efficiency of ADCC [118,119]. Similarly, sialylation (the addition of sialic acid) also enhances anti-inflammatory activity and reduces the affinity of IgG Fc for FcγRs [120,121,122]. Furthermore, a terminal galactose has been shown to increase ADCC, CDC and ADCP [123,124,125]. In the context of HIV-1, a recent study has reported significant differences in the overall IgG glycosylation profiles depending on the disease status of HIV-1-infected individuals, with elite controllers harboring more non-fucosylated and less sialylated glycoforms compared to chronic progressors [126]. Additionally, non-fucosylated and galactosylated antibodies were shown to negatively correlate with levels of cell-associated HIV DNA and RNA in antiretroviral therapy (ART)-suppressed individuals [127]. Additional studies to understand the impact of Fc-glycosylation against virally infected cells are warranted, since a non-human primate study using a non-fucosylated b12 did not show enhanced protection from SHIV challenges, as compared to wild type b12 [128]. Furthermore, a study by Li et al. showed that sialylated Fc IgGs and their diminished affinity for FcγRs in vitro do not impact outcomes in vivo [129], where sialylation was shown to only significantly decrease ADCC in the presence of fucosylation and did not have an impact alone.

Current research to enhance the therapeutic activity of IgGs and antibody-mediated effector functions against viral infections is focusing on Fc-glycosylation engineering. The N-glycans of IgG proteins can be customized by using a biologic medium adaptable to glycoengineering, such as *Nicotiana benthamiana* [130]. Antibody glycoengineering has been exploited to improve the efficacy of bNAb–FcγR interactions against HIV-1-infected cells. This was accomplished using a 2G12 glycoform lacking the core fucose and plant-specific xylose to enhance antibody-dependent cell-mediated virus inhibition against HIV-1, along with significantly enhanced FcγRIIIa binding [131]. Similar modifications were carried out for the bNAbs VRC01 and PG9, where the fucose- and xylose-free glycoforms had significantly higher affinities to form stable complexes with FcγRs and induce significantly higher ADCC against infected cells, respectively [132,133]. In terms of other viral infections, glycoengineered mAbs produced in plants have been incorporated into the ZMapp cocktail to improve efficacy against EboV [107]. Moreover, a non-fucosylated and non-galactosylated glycovariant of the Palivizumab mAb against RSV showed improved in vivo protective capacity by decreasing viral titers, despite comparable neutralization capacity [134]. Finally, antibody glycoforms produced in *N. benthamiana* and lacking xylose and fucose are also being developed as candidates to treat West Nile virus [135], dengue virus [136], and CHIKV [137] infections. As we have discussed above, the glycosylation heterogeneity of the Fc portion of the antibody has dramatic impacts on Fc-mediated effector functions. The generation of antibodies with near homogenous glycosylation will facilitate the reproducibility among studies and the generation of new therapeutics.

## 6. Conclusions

It is becoming increasingly clear that several factors contribute to the efficacy of Fc-mediated effector responses against virally infected cells. The conformation of Env being recognized by any given antibody, its rate of internalization, the affinity of the antibodies for different FcγRs, Fc receptor polymorphism, the modulation of stress ligands by the virus, and the presence or not of soluble forms of Env are just a few examples of an expanding number of parameters governing effector functions. A better understanding of all these parameters is required to further improve antibodies for therapeutic and vaccine designs.
